# Understanding Life at High Temperatures: Relationships of Molecular Channels in Enzymes of Methanogenic Archaea and Their Growth Temperatures

**DOI:** 10.3390/ijms232315149

**Published:** 2022-12-02

**Authors:** Laura F. Ginsbach, Juan M. Gonzalez

**Affiliations:** 1 Institute of Biotechnology, Department of Applied Biochemistry, Technische Universität Berlin, 10623 Berlin, Germany; 2 Instituto de Recursos Naturales y Agrobiología de Sevilla, Consejo Superior de Investigaciones Científicas, IRNAS-CSIC, 41012 Sevilla, Spain

**Keywords:** molecular channels, molecular tunnels, hyperthermophiles, thermal stability, methanogen, Archaea

## Abstract

Analyses of protein structures have shown the existence of molecular channels in enzymes from Prokaryotes. Those molecular channels suggest a critical role of spatial voids in proteins, above all, in those enzymes functioning under high temperature. It is expected that these spaces within the protein structure are required to access the active site and to maximize availability and thermal stability of their substrates and cofactors. Interestingly, numerous substrates and cofactors have been reported to be highly temperature-sensitive biomolecules. Methanogens represent a singular phylogenetic group of Archaea that performs anaerobic respiration producing methane during growth. Methanogens inhabit a variety of environments including the full range of temperatures for the known living forms. Herein, we carry out a dimensional analysis of molecular tunnels in key enzymes of the methanogenic pathway from methanogenic Archaea growing optimally over a broad temperature range. We aim to determine whether the dimensions of the molecular tunnels are critical for those enzymes from thermophiles. Results showed that at increasing growth temperature the dimensions of molecular tunnels in the enzymes methyl-coenzyme M reductase and heterodisulfide reductase become increasingly restrictive and present strict limits at the highest growth temperatures, i.e., for hyperthermophilic methanogens. However, growth at lower temperature allows a wide dimensional range for the molecular spaces in these enzymes. This is in agreement with previous suggestions on a potential major role of molecular tunnels to maintain biomolecule stability and activity of some enzymes in microorganisms growing at high temperatures. These results contribute to better understand archaeal growth at high temperatures. Furthermore, an optimization of the dimensions of molecular tunnels would represent an important adaptation required to maintain the activity of key enzymes of the methanogenic pathway for those methanogens growing optimally at high temperatures.

## 1. Introduction

Protein channels or tunnels play critical roles for diverse enzymes such as in the mobility of ligands to buried active sites, in channeling intermediates through multiple steps of metabolic pathways, or in the transport of different molecules (substrate and product) and ions. The selective nature of each channel allows the access of specific molecules and create a microenvironment essential for these molecules [1,2]. It has been shown that changes altering the molecular channels result in missing functionality [2,3].

In spite of recent developments on deciphering the structure and properties of proteins (i.e., enzymes), scarce information is available on the role of void spaces within protein structures and their influence on protein activity and stability above all in the Archaea. Molecular channels, tunnels, and pores all represent geometrically an empty space within an internal space of a molecule (e.g., the active site of a protein) with the molecular exterior or a pass through that molecule. Herein, we include all these geometries in the terms molecular channels or tunnels. Molecular tunnels or channels have been reported to be intimately related to the biological function and structural stability of proteins [4,5,6]. The access of substrates and cofactors to the active site of enzymes frequently occurs through internal passages connecting exterior to interior protein spaces, as well as allowing the release of the generated reaction products [2]. Channels (generally above 15 Å long) are common features in the structure of many enzymes and they have been reported in 64% of deciphered protein crystal structures [7], suggesting a relevant role in their functionality.

Several potential aspects of relevance for molecular channels have been suggested [1,8,9]. Passage of substrates, intermediates, and products via molecular channels reduces the transit time to or from the active centers and facilitates an efficient transference within multifunctional enzymes or enzymatic complexes. Ligand transport, molecular docking trajectories, and the potential of molecular tunnels and channels in biotechnology have been recently analyzed [10,11]. Holding substrates in protein empty spaces can ensure substrate availability and loss of reaction intermediates by diffusion, thus increasing reaction efficiency. Isolating substrates, intermediates, or products within the protein internal tunnels can avoid potential effects of inhibition or reduced rates for some cellular processes or other enzymatic reactions [2]. Molecular tunnels can be used to distribute specific substrates, intermediates, or products along a multi-enzymatic chain of reactions or metabolic pathways. This would contribute to accelerate the catalytic steps to progress along the pathway, increasing efficiency. Furthermore, proteins by molecular channeling can chaperone labile substrates and intermediates, increasing their stability within the protein structure [2,12]. The actual role of molecular tunnels on specific processes remains, in most cases, to be analyzed in detail although their potential general contributions have been suggested [1,7,8,9].

Multiple reports have shown that a number of cellular and molecular features can maintain the molecular stability of proteins, nucleic acids, and lipids [13,14,15]. However, how thermophiles and hyperthermophiles can thrive under high temperatures remains to be fully understood. For instance, some thermolabile biomolecules (ATP, NAD/H, NADP/H, pyridoxal phosphate, etc.) are known to be key components of the metabolism in all living forms including Bacteria and Archaea [16,17]. A question to be solved is how thermophiles can live at high temperatures using mostly the same universal biomolecules than all other organisms [18,19]. A simple strategy to allow the growth of thermophiles maintaining the thermal stability of labile biomolecules was recently proposed by Cuecas et al. [12]. These authors concluded that maintaining intracellular viscosity can provide thermal stability to some low-molecular-weight biomolecules although this mechanism appeared to be inefficient above 80 °C [10]. Thus, hyperthermophiles might require additional adaptations and some complementary alternatives have been proposed [12,16,17]. Among them, molecular channeling could be a feasible possibility to preserve the stability of labile biomolecules against high temperatures. The relevance of this mechanism in hyperthermophiles has been pointed out [20] for a couple of enzymes related to amino-acid metabolism: glutamate dehydrogenase and glutamine phosphoribosyl pyrophosphate amidotransferase. However, no information is available on the relationship of optimum growth temperature and the requirements for optimized molecular tunnels in other metabolic pathways and microorganisms.

The enzymes heterodisulfide reductase (Hdr) and methyl-coenzyme M reductase (Mmr) are involved in the last step of the three major methanogenic pathways [21,22,23]; therefore, they were selected for this study because they are common to all methanogens.

Methanogens represent a singular phylogenetic group of Archaea with the differential characteristic of producing methane as a final product of their metabolism. Methane is an important gas which can be used as biofuel [21,22]. In recent years, methane has attracted particular interest because it is a major greenhouse gas emitted to the atmosphere. Methane is mainly produced biologically, and this singular process is carried out by methanogenic Archaea [23]. Methanogens are widely distributed in oxygen-free environments and have been reported to grow at temperatures ranging from below 0 °C [24] up to 122 °C [25] and all temperatures in between [21]. As a result of the singularity of this group of Archaea and their typical metabolism (i.e., the methanogenic pathway), it could be an ideal model to analyze the dimensions and potential relevance of molecular tunnels in the key and common enzymes involved in the methanogenic pathway over a broad range of temperatures for growth. 

This study aims to infer if the molecular tunnels and their dimensions present a critical contribution to growth of methanogenic Archaea at high temperatures by analyzing key enzymes of the methanogenic pathway. A comparative analysis was performed including the broadest spectrum range of growth temperatures. The study was focused on realizing whether the dimensions of the molecular tunnels for hyperthermophilic methanogenic Archaea present relatively more or less relevance than for the low temperature methanogens. Optimized molecular tunnel dimensions should imply a restricted variability of these tunnel dimensions and that is what one should expected in high-temperature growing methanogenic Archaea when compared to low-temperature growing methanogens.

## 2. Results

**Figure 1:**
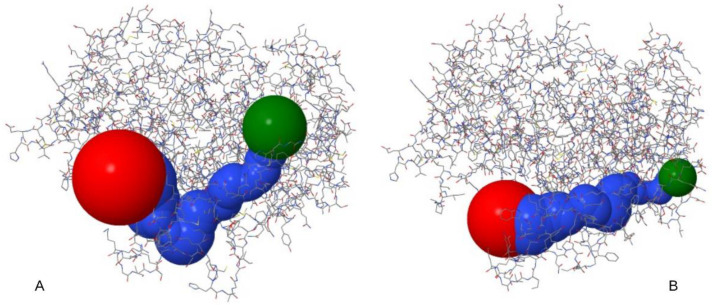
An example of the visualization of a predicted molecular tunnel in methyl-coenzyme M reductase (alpha subunit) from *Methanomicrobium mobile* ((**A**); 37 °C optimum growth temperature) and *Methanopyrus* sp. KOL6 ((**B**); 110 °C optimum growth temperature).

A comparative example of the visualization of predicted molecular tunnels in methyl-coenzyme M reductase (alpha subunit) from *Methanomicrobium mobile* (37 °C optimum growth temperature) and *Methanopyrus* sp. KOL6 (110 °C optimum growth temperature) is shown in Figure 1.

**Figure 2:**
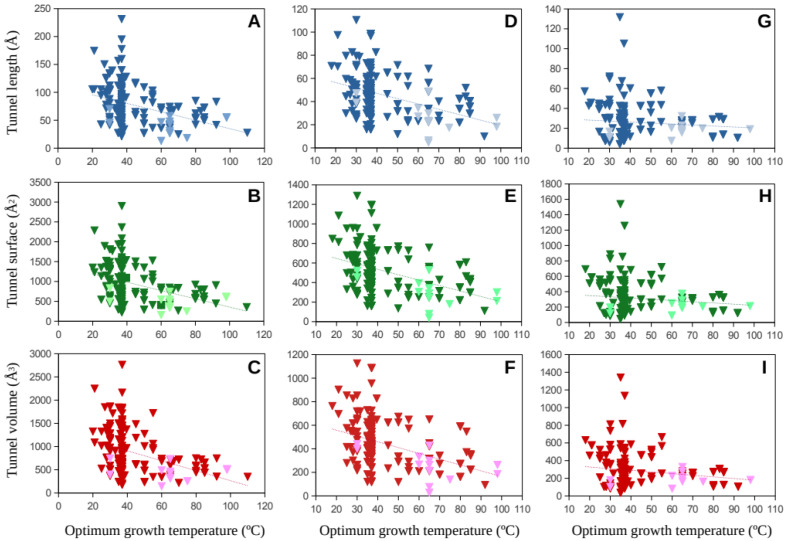
Molecular tunnel dimensions, length (**A**,**D**,**G**), surface (**B**,**E**,**H**), and volume (**C**,**F**,**I**) (in Å), predicted for the methyl-coenzyme M reductase (Mmr) subunit genes, alpha (**A**,**B**,**C**), beta (**D**,**E**,**F**), and gamma (**G**,**H**,**I**), from numerous methanogenic Archaea as a function of their optimum growth rates. Dark color-filled symbols correspond to predicted protein structures. Light color-filled symbols correspond to resolved model structures from the Protein Data Bank. Trend lines are presented as dotted lines.

**Figure 3:**
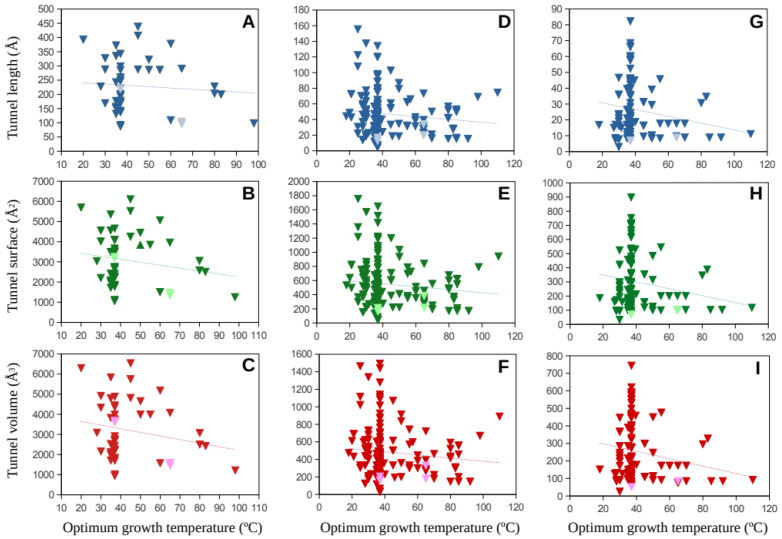
Molecular tunnel dimensions, length (**A**,**D**,**G**), surface (**B**,**E**,**H**), and volume (**C**,**F**,**I**) (in Å), predicted for the heterodisulfide reductase (Hdr) subunit genes, A (**A**,**B**,**C**), B (**D**,**E**,**F**), and C (**G**,**H**,**I**), from numerous methanogenic Archaea as a function of their optimum growth rates. Dark color-filled symbols correspond to predicted protein structures. Light color-filled symbols correspond to resolved model structures from the Protein Data Bank. Trend lines are presented as dotted lines.

Molecular tunnel length, surface, and volume estimates for the major subunits of the Mmr and Hdr proteins were plotted against the optimum growth temperature for methanogenic Archaea covering a wide range of temperatures (Figure 2 and Figure 3). The numbers of Mmr sequences analyzed were 179, 183, and 166 from the alpha, beta, and gamma subunits, respectively. The total of sequences processed for the Hdr sequences was: 52, 159 and 107 from the A, B and C subunits, respectively. It is worth noting that different subunit genes can present completely different tunnel/channel dimensions because their amino-acid sequences and structures are different, and this also depends on the specific function of each subunit.

Results show a wide distribution of the tunnel dimensions in the low-temperature range (mesophilic temperature range) whereas the estimated dimensions tend to point toward a narrower range at high temperatures. This is observed for all the studied cases (Figure 2 and Figure 3). Among the predicted 3D protein structures, a number of resolved structures were included as comparison to infer potential additional noise due to protein modeling. Generally, resolved proteins fit well in the distribution of the predicted protein structures.

**Figure 4:**
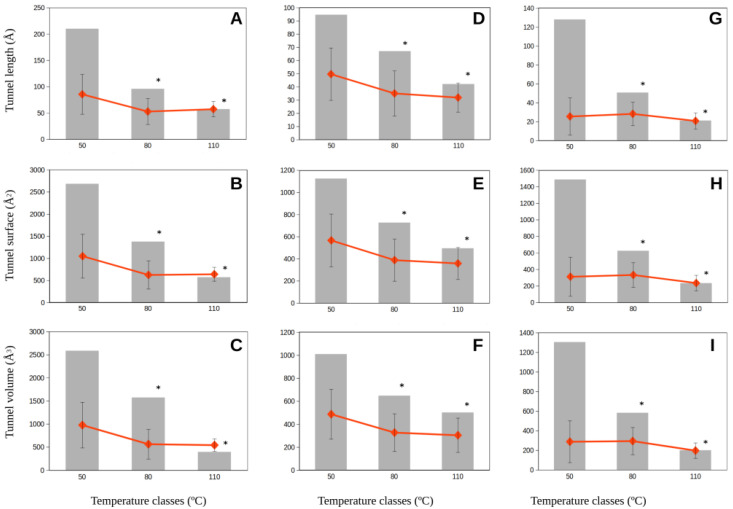
Amplitude and average of the molecular tunnel dimensions, length (**A**,**D**,**G**), surface (**B**,**E**,**H),** and volume (**C**,**F**,**I**) (in Å), predicted for the methyl-coenzyme M reductase (Mmr) subunit genes, alpha (**A**,**B**,**C**), beta (**D**,**E**,**F**), and gamma (**G**,**H**,**I**) from methanogenic Archaea classified in three classes of optimum growth temperature (<50 °C; 50–80 °C; >80 °C). Gray bars correspond to the amplitude of data observed for estimates of the molecular tunnel dimensions. Red squares represent the average dimensions for each growth temperature class. Error bars represent the standard deviation around the average values. Asterisks denote statistical significance (*p* < 0.05) with respect to the temperature class <50 °C.

**Figure 5:**
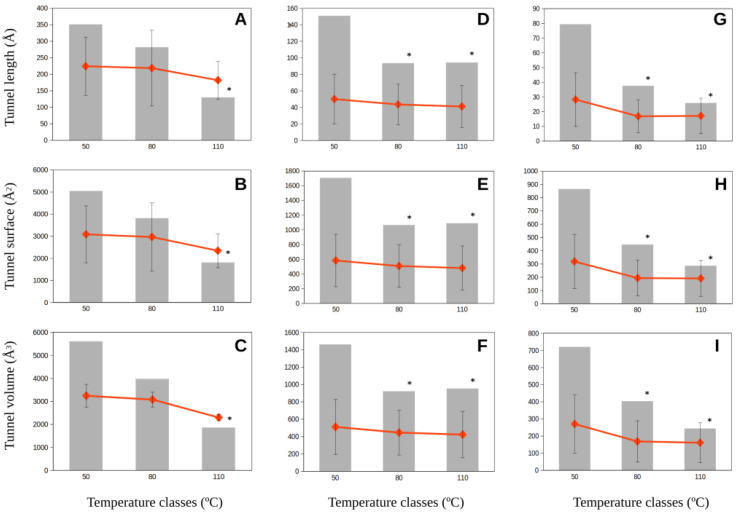
Amplitude and average of the molecular tunnel dimensions, length (**A**,**D**,**G**), surface (**B**,**E**,**H),** and volume (**C**,**F**,**I**) (in Å), predicted for the heterodisulfide reductas (Hdr) subunit genes, A (**A**,**B**,**C**), B (**D**,**E**,**F**), and C (**G**,**H**,**I**) from methanogenic Archaea classified in three classes of optimum growth temperature (<50 °C; 50–80 °C; >80 °C). Gray bars correspond to the amplitude of data observed for estimates of the molecular tunnel dimensions. Red squares represent the average dimensions for each growth temperature class. Error bars for the average values represent a standard deviation. Asterisks denote statistical significance (*p* < 0.05) with respect to the temperature class < 50 °C.

If the amplitudes of the variability of molecular tunnel estimates were considered in growth temperature classes, we observed that the amplitude of molecular tunnel dimensions decreased with increasing optimum growth temperatures for the studied proteins from methanogenic Archaea (Figure 4 and Figure 5). Thus, the amplitudes of the estimates at the lowest class of growth temperatures (<50 °C) were 2–7-fold larger than those for methanogens in the highest growth temperature class (>80 °C) (Figure 4 and Figure 5). These relevant changes in how permissive the molecular tunnel dimensions (length, surface, and volume) are for mesophilic methanogenic Archaea contrast the much more restrictive amplitude on these estimates from hyperthermophilic methanogens. Nevertheless, we observed no significant differences among the average of those molecular tunnel dimensions because the values estimated for the lowest growth temperature class (<50 °C) included the estimates for the highest growth temperature class (>80 °C) (Figure 4 and Figure 5). The enzymes corresponding to the highest growth temperature class (>80 °C) showed a more restrictive range of variability than the broad variability range observed for enzymes from mesophilic methanogens.

## 3. Discussion

Understanding how thermophiles can inhabit exclusively under high-temperature conditions is a point that requires further investigation. Although most macromolecules (proteins, nucleic acids, etc.) have been studied in depth and their response and adaptations required to function under high temperatures are relatively well understood [14,16], the case for small biomolecules remains to be solved. A number of universal biomolecules are known to be thermolabile such as NADH and ATP [16,17,20]. Recently, Cuecas et al. [12] showed that maintaining intracellular viscosity contributes significantly to stabilizing some biomolecules (i.e., NADH), although this strategy did not look feasible for the hyperthermophiles grown above 80 °C. Previous reviews have pointed out the possibility that mechanisms of molecular channeling could contribute to increase the thermal stability of labile biomolecules. Gonzalez [20] pointed out a restriction of the dimensions of molecular spaces in glutamate dehydrogenase and glutamine phosphoribosyl pyrophosphate amidotransferase for hyperthermophiles unlike the mesophiles. Herein, we focus on key enzymes of the methanogenic pathway because the methanogens, a functionally singular group of Archaea, present representatives over a very broad range of optimum growth temperatures and represent a good phylogenetic group of microorganisms to test the proposal that the dimensions of molecular tunnels in the protein structure are restricted for hyperthermophiles, whereas the mesophiles do not present those restrictions in their protein structures.

The strategy of imposing restrictive limits to the molecular channels in methanogens growing at high temperatures presents various explanations. First, tighter molecular channels should result in more compact proteins, which results in facilitating the interaction among amino acid residues by reducing the length between potential interacting residues. The existence of more compact proteins in hyperthermophiles has been previously proposed [14,20,26,27]. The cases of glutamate dehydrogenase and glutamine phosphoribosyl pyrophosphate amidotransferase, enzymes that show a reduction in open spaces in the protein 3D structure of microorganisms growing optimally at high temperature, have suggested that thermophily imposes adaptive mechanisms to allow growth at high temperatures by limiting the dimensions of the molecular channels [20]. 

Numerous enzymes require cofactors and substrates [4] that are labile to high temperatures. Thus, in order for these enzymes to work properly, a concentration of those biomolecules (i.e., cofactors and substrates) needs to be maintained. Adequately dimensioned molecular channels must be an adaptation for the proper functioning of these enzymes. Furthermore, it has been reported that molecular channeling is a proven strategy for enhancing the availability of required biomolecules (cofactors and additional substrates), increasing stability and reducing enzymatic competence within complex pathways, for numerous enzymes [2,7,12]. This can be proposed for the case of methanogens growing at high temperatures when thermolabile biomolecules are scarce or present high demand for numerous enzymes, similarly to previous suggestions on glutamine phosphoribosylpyrophosphate amidotransferase from hyperthermophilic Bacteria and Archaea [20], along with many other examples from other organisms [9]. Hyperthermophilic methanogenic Archaea have adapted to high temperatures by showing compact proteins that maintain the dimensions of molecular tunnels within a narrow range for optimum performance, stability, and overall functionality at high temperatures.

In spite of our lack of understanding of the actual strategies and mechanisms that rule growth at high temperatures for the hyperthermophiles, the fact that these organisms grow optimally under high temperatures, using basically the same biomolecules as all other living beings, confirms the capability of hyperthermophiles to maintain the stability at high temperatures for universal, thermolabile low-molecular-weight biomolecules (e.g., NADH, NADPH, ATP, and pyridoxal phosphate). Optimally dimensioned molecular channels can benefit hyperthermophiles through various mechanisms [12,16,17]. For example, molecular channeling could maintain the stability of small biomolecules by enhancing molecular interactions. In addition, these channels could preserve adequate stocks and transfer these labile biomolecules and cofactors directly to the enzyme active sites. Further investigation will focus on fully understanding the relevance of molecular tunnels on the capability of hyperthermophiles to inhabit high-temperature environments. Research should center on the experimental determination of molecular interactions representing a structural mechanism required for optimal growth at the upper temperature range for life.

## 4. Materials and Methods

Key enzymes of the methanogenic pathway were analyzed: methyl-coenzyme M reductase (Mmr) and heterodisulfide reductase (Hdr). Because the methanogenic pathway represents an essential metabolic step for methanogens [28], these enzymes need to be optimized to function properly under the cell growth conditions. Amino-acid sequences for these enzymes from microorganisms covering the whole temperature range for growth of methanogenic Archaea were downloaded from NCBI (National Center for Biotechnology Information; https://www.ncbi.nlm.nih.gov/ (accessed on 30 November 2022)). Optimum growth temperatures were retrieved from https://bacdive.dsmz.de/ (accessed on 30 November 2022). The 3D-structures for these proteins were predicted by Phyre2 [29]. These proteins corresponded to Archaea presenting optimum growth temperatures in the range from 18 °C to 110 °C (Supplementary Materials). Further analyses were carried out basically as previously reported [20] and briefly described below. The software MOLE [30] was used to identify and localize the tunnels from the predicted structures of each protein. The MOLE strategy to explore molecular voids is based on Voronoi diagrams [30] overcoming some limitations of other software algorithms including errors due to grid extrapolation and excessive computer demands. MOLE results provide the localization and dimensions of molecular channels; from that information, the total channel length, lateral surface, and volume were calculated. The total length, lateral surface, and volume of the channels in a protein were the sums of the sections identified and localized in that protein. For each section of the tunnels, lateral surface (S) and volume (V) were calculated assuming the shape of a cylinder or truncated cone using the following formulas:

V = (1/3) π h (r_1_^2^ + r_1_ r_2_ + r_2_^2^),

$$S=\pi \;(r_{1}+r_{2})\sqrt{\left[{\left(r_{1}-r_{2}\right)}^{2}{+h}^{2}\right]},$$

where r_1_ and r_2_ are the two radii of the truncated cone, and h represents its height. In the case of a cylinder, r_1_ and r_2_ are equal. To show some examples of molecular tunnels (i.e., Figure 1), they were visualized using the software tool CHEXVIS [31].

Available model structures from the studied enzymes were included in the analyses for comparative purposes as a reference for the results of the structures predicted from their amino-acid sequences (Supplementary Materials). Model structures were obtained from the Protein Data Bank (PDB; https://www.rcsb.org (accessed on 30 November 2022)). A list of the sequences analyzed in this study and the model structures is available in the Supplementary Materials, showing their accession numbers, optimum growth temperatures, and species names. Trend lines were estimated by linear regression [32]. The amplitude of values for the dimensions of molecular tunnels at a growth temperature class was calculated as the difference between its maximum to its minimum values, which represents the variability existing in each temperature class. The significance of differences in that variability was determined from the critical values of the F-distribution [32], and multiple testing correction by Bonferoni adjustment was applied.

## Data Availability

Data corresponding to Figure 2, Figure 3, Figure 4 and Figure 5 are available at https://digital.csic.es/ (accessed on 30 November 2022) at the links https://hdl.handle.net/10261/281573 (accessed on 30 November 2022) (Figure 2), https://hdl.handle.net/10261/281568 (accessed on 30 November 2022) (Figure 3), and https://hdl.handle.net/10261/281562 (accessed on 30 November 2022) (Figure 4 and Figure 5).

## Supplementary Materials

The following supporting information can be downloaded at https://www.mdpi.com/article/10.3390/ijms232315149/s1.
